# Primary Cilia in the Skin: Functions in Immunity and Therapeutic Potential

**DOI:** 10.3389/fcell.2021.621318

**Published:** 2021-02-11

**Authors:** Manami Toriyama, Ken J. Ishii

**Affiliations:** ^1^Graduate School of Pharmacological Sciences, Osaka University, Osaka, Japan; ^2^Center for Vaccine and Adjuvant Research, National Institutes of Biomedical Innovation, Health and Nutrition, Osaka, Japan; ^3^Graduate School of Science and Technology, Nara Institute of Science and Technology, Nara, Japan; ^4^Laboratory of Vaccine Science, World Premier International Research Center Initiative (WPI) Immunology Frontier Research Center, Osaka University, Osaka, Japan; ^5^Division of Vaccine Science, The Institute of Medical Science, The University of Tokyo, Tokyo, Japan

**Keywords:** primary cilia, immunity, epidermis, atopic dermatitis, keratinocyte, Langerhans cell

## Abstract

The skin is the biggest organ and provides a physical and immunological barrier against pathogen infection. The distribution of primary cilia in the skin of mice has been reported, but which cells in human skin have them has not, and we still know very little about how they change in response to immune reactions or disease. This review introduces several studies that describe mechanisms of cilia regulation by immune reaction and the physiological relevance of cilia regulating proliferation and differentiation of stroma cells, including skin-resident Langerhans cells. We discuss the possibility of primary cilia pathology in allergic atopic dermatitis and the potential for therapies targeting primary cilia signaling.

## Introduction

Kowalevsky (1867) first reported the presence of non-motile cilia (primary cilia) in a variety of vertebrate cells. For a long time, many scientists thought that non-motile cilia were a non-functional vestigial organ. However, almost 150 years after the first finding of non-motile cilia, they are now recognized as a sensory organelle involving hearing, sight, and other sensory input. A primary cilium is a unique organelle protruding from the cell surface. Various receptors, including G protein–coupled receptor (GPCR) and tyrosine kinase type receptor, and ion channels localized in primary cilia enable cells to sense the extracellular environment and transduce signals inside the cell that control cell function (Phua et al., 2015; Schou et al., 2015; Christensen et al., 2017). It is widely accepted that almost all cell types can have primary cilia, including skin cells.

**FIGURE 1:**
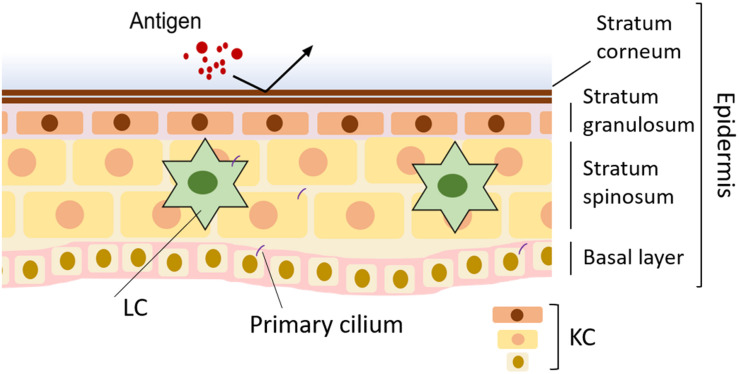
Normal skin and disruption of primary cilia in skin disease. In normal skin, the barrier formed by keratinocytes (KCs) blocks infection. Langerhans cells (LCs) in the epidermis contribute to skin homeostasis by working as antigen-presenting cells.

The skin is the heaviest organ in the human body and, including the subcutaneous tissue, accounts for 16% of body weight (Shimizu, 2018). The skin defines the body and maintains homeostasis by preventing water loss, regulating body temperature, and sensing mechanical stimuli. It also functions as an immune organ that prevents foreign materials from invading from outside (Shimizu, 2018; Figure 1A). The skin has a three-layer structure: epidermis, dermis, and hypodermis—each with its own anatomy and function (Wong et al., 2016; Shimizu, 2018). The epidermis is 0.2 mm thick, and about 90% of epidermal cells are keratinocytes (KCs). The most important role of KCs is forming a skin barrier. Both the physical barrier formed by KCs and the immune barrier composed of immune cells prevent invasion of pathogens (Eyerich et al., 2018; Kabashima et al., 2019; Figure 1A).

Coordinated differentiation and proliferation of KCs—namely, keratinization—are required for barrier formation and are strictly regulated depending on the extracellular environment, such as cell density (Eckert et al., 2002; Simpson et al., 2011; Roshan et al., 2016). Most keratinocyte stem cells (KSCs) in the epidermal basal layer continue to proliferate randomly, and some produce transitory amplifying cells (TAs) by asymmetric division (Mackenzie, 1970; Clayton et al., 2007). The TAs, with a much higher proliferative capacity than KSCs, maintain the stem cell pool (Hsu et al., 2014). Daughter cells produced by immature KCs asymmetric division of both KSCs and TAs differentiate by stimuli that are not yet known and then mature with migration from the basal layer (Lechler and Fuchs, 2005; Mascré et al., 2012). Terminally differentiated KCs die and form a cornified layer consisting of keratin and a cornified envelope (Candi et al., 2005). The cornified envelope is formed from the plasma membrane of mature KCs and consists of involucrin and loricrin. Both keratin and the cornified envelope work as a physical barrier against water loss, damage by UV radiation, and pathogen infection. Barrier disruption increases the risk of ichthyosis and atopic dermatitis (AD) (Herrmann et al., 2003; Palmer et al., 2006; Klar et al., 2009; Rerknimitr et al., 2017; Yamamoto et al., 2020).

Moisturization is widely accepted to reduce the risk of AD because barrier dysfunction causes dryness, which activates peripheral neurons to induce severe itching and scratching, which exacerbates the disease. We know that the pathology of AD is complicated and the full picture of its pathophysiology is not clear, so symptomatic treatment is often used, typically topical steroids and immunosuppressants in parallel with moisturizer (Eichenfield et al., 2014a; Katoh et al., 2019).

Destruction of the skin barrier allows pathogens to invade in the body, where they induce an immune response (De Benedetto et al., 2012). When Langerhans cells (LCs), a type of dendritic cell (DC), in the epidermis take up pathogens, they become fully functional and move to the lymph nodes, where they present the antigens to T cells (Nestle et al., 2009; Pasparakis et al., 2014; Deckers et al., 2018). Thus, LCs play an important role in bridging innate immunity and acquired immunity. LCs are present at a rate of about 2–5% in normal epidermis but increase in AD: 13–16% of KCs isolated from AD patients *in vitro*, and nearly 30% *in vivo*, were positively stained with a proliferation marker, ki67 (Czernielewski and Demarchez, 1987; Chorro et al., 2009; Toriyama et al., 2020). The elimination of LCs suppressed the immune response induced by protein antigens (Nakajima et al., 2012). Thus, good control of the count, proliferation, and maturation of LCs may help to regulate immune responses and thus treat AD.

It is very difficult to understand the pathophysiology of human inflammatory diseases because mechanisms of inflammatory responses are so complicated, and the immune response between model rodents and humans are usually different (Beura et al., 2016). Recently, we showed that the primary cilium, which acts as a signaling hub, regulates the proliferation, differentiation, and maturation of human KCs and LCs (Toriyama et al., 2020). This review discusses the function of primary cilia, especially inflammatory regulation, in human skin in the context of the literature, and the possible therapeutic potential of targeting signaling pathways transduced in primary cilia.

## Primary Cilia Distribution and Functions in the Skin

The primary cilium is a unique organelle that plays an important role in transducing extracellular signals into the cytosol. Since skin is the outermost organ covering the body, it must be able to sense the extracellular environment, suggesting a role of primary cilia in it. Primary cilia have been identified in several skin-resident cells: human/mouse KC and basal cell carcinoma (BCC) (Elofsson et al., 1984; Strugnell et al., 1996; Wong et al., 2009; Ezratty et al., 2011; Toriyama et al., 2020), human/mouse fibroblast (Strugnell et al., 1996; Toriyama et al., 2020), human melanocyte (Elofsson et al., 1981; Choi et al., 2016), and LC (Toriyama et al., 2020).

Functions of primary cilia in KCs have been reported. In mouse embryonic epidermis, inhibition of primary cilia formation by shRNA targeting *ift74* and *ift88* caused hyperproliferation of KCs, whereas inhibition by *kif3A* knockdown inhibited cell growth while inhibiting primary cilia formation (Ezratty et al., 2011). Overexpression of polo-like-kinase4 increased centrosome number, primary cilia disruption, and keratin5-positive proliferating KC, with decreasing KC differentiation markers, including involucrin, filaggrin, and loricrin (Coelho et al., 2015). In contrast, both primary cilia counts and ki67-positive KCs in AD patients were significantly increased relative to healthy epidermis, and there was a relation between primary cilia increase and loricrin decrease, but not filaggrin or IgE level (Toriyama et al., 2020). These findings suggest that primary cilia both promote KC proliferation and inhibit maturation in allergic conditions and raise the question of what signaling is transduced in primary cilia to regulate KC proliferation or maturation in inflammatory conditions. Ezratty et al. (2011) strongly suggested that downregulation of *ift74*, or chloral hydrate treatment to diminish primary cilia, inhibited the notch signaling pathway, which is transduced in primary cilia, and decreased the expression of a KC maturation marker, K10. Another report suggested that proliferation of KC was promoted by PDGF-AA, the receptor for which, PDGFRα, is specifically localized in primary cilia (Toriyama et al., 2020). Thus, understanding KC biology is complicated by the lack of knowledge of molecular mechanisms by which proliferation and differentiation signals via primary cilia are regulated spatiotemporally. Recently, presenilin and arf4 have been identified as KC differentiation regulators (Ezratty et al., 2016). These are localized in the basal body and are thought to regulate K10 expression, but they might not control cilia formation directly (Ezratty et al., 2016). So, understanding how they regulate notch signaling will provide insight into KC differentiation and maturation. Future analysis of tissue microenvironments, including concentrations of extracellular molecules, tissue dependency, and age dependency, and investigation of pathophysiological conditions will explain primary cilia regulation and signaling.

What is the skin phenotype when primary cilia are inactivated? PCP effector gene, *fuzzy*, knockout or specific knockout of *ift88* in adult KCs by K14 promoter induced ventral alopecia, basaloid hyperplasia, epidermal ingrowth, disorganized hair follicles, and excess sebaceous gland lobules, with accumulating ΔNp63 transcription factor (Croyle et al., 2011; Dai et al., 2011). Coincident with primary cilia disruption in the epidermis, *ift88* or *kif3a* conditional knockout in the ventral dermis of prx1-cre mice caused severe hypotrichosis with downregulation of sonic hedgehog (shh) signaling (Lehman et al., 2009). Molecular mechanisms by which primary cilia regulate hair growth have been reported: laminin-511-β1 integrin signaling promoted primary cilia formation in dermal papillae, which is required for shh and PDGFRα signaling to develop hair (Gao et al., 2008). These results suggest that primary cilia in epidermal KCs, dermal papillae, and fibroblasts are indispensable for hair growth and that primary cilia are important for skin homeostasis.

Some functions of primary cilia in melanocytes have been reported. The use of “smoothened agonist” to activate shh signaling inhibited melanogenesis, whereas the use of specific cytoplasmic dynein inhibitor, ciliobrevin A1, to inhibit primary cilia formation promoted melanogenesis (Choi et al., 2016). In melanoma, primary cilia were deconstructed, while EZH2, a unit of PRC2 methyltransferase, induced primary cilia loss by silencing genes involved in primary cilia formation, which drove metastatic melanoma (Zingg et al., 2018). Since the loss of primary cilia in melanoma induced the activation of the Wnt/β-catenin pathway (Zingg et al., 2018), regulation of this pathway via primary cilia formation may contribute to the treatment of cancer and pigmentation disorder.

## Immunocompetent Cells in Epidermis and Their Functions

**FIGURE 2:**
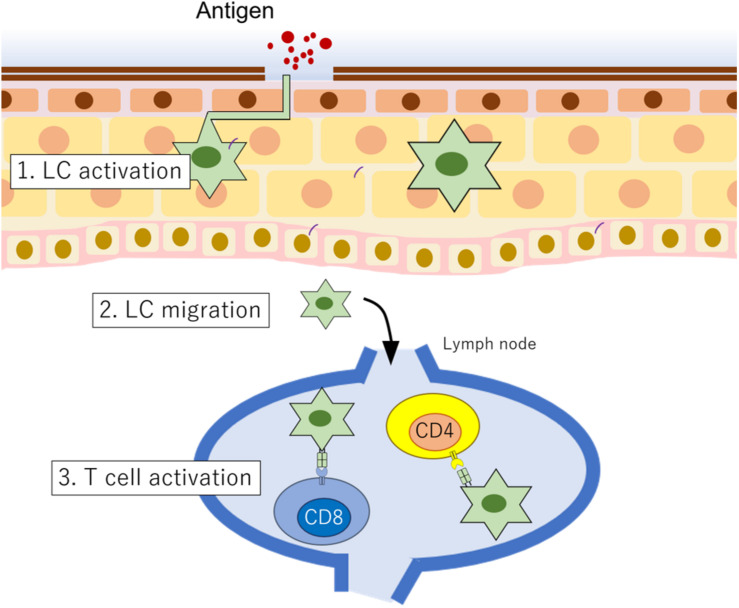
LC function in epidermis. When LCs incorporate antigens, (1) they are rapidly functionally activated, and then (2) they migrate to the lymph node. (3) In the lymph node, LCs interact with T cells to present antigens.

Many types of cells are involved in inflammatory responses in skin diseases. In this section, we summarize and discuss the functions of LCs and KCs. Langerhans (1868) first found and described LCs. From their morphology, LCs were first interpreted as a kind of neuronal cell, not as immune cells. For almost 100 years, their function was unknown. However, Stingl et al. (1977) and Klareskog et al. (1977) identified that LCs express MHC class II molecules and macrophage antigens, and Schuler and Steinman (1985) found that they have a role as APCs. LCs are the sole APCs in the epidermis. When they incorporate microbial antigens, they rapidly mature, and as functionally activated APCs, they migrate to the lymph nodes, interact with T cells via MHC class II molecules, and display the antigen to the T cells. Thus, LCs act as a bridge between innate immunity and acquired immunity (Nestle et al., 2009; Pasparakis et al., 2014; Deckers et al., 2018; Figure 2). Interestingly, activated LCs extend their dendrites through tight junctions formed by KCs (Kubo et al., 2009). This fact strongly suggests that activated LCs actively take antigens and probably present them continuously to T cells to maintain skin homeostasis.

Recently, to elucidate the role of LCs in antigen presentation and in disease, researchers generated genetically modified mice in which LCs can be specifically removed (Bennett et al., 2005; Kaplan et al., 2005; Kissenpfennig and Malissen, 2006; Honda et al., 2010). In a series of elegant experiments, Honda et al. (2010) indicated that LCs are not essential to the development of contact hypersensitivity caused by small molecules like dinitrofluorobenzene, because LC-depleted mice swabbed with hapten (MW <1,000) did not have the decreased immune reactivity found in contact dermatitis. In contrast, LC-depleted mice swabbed with protein antigens of MW >5,000 had inhibited skin inflammation (Nakajima et al., 2012). These analyses strongly suggest that LCs are involved in the development of AD caused by protein antigens but not in contact hypersensitivity induced by small molecules. Thus, LCs especially recognize protein antigens and play an important role in maintaining skin homeostasis.

Not only do KCs form a physical barrier, but they also produce cytokines in response to immune stimulation and thus confer skin immunity. The secretion of cytokines by KCs, their effects, and their secretion mechanism have been studied for decades (see reviews Hänel et al., 2013; Noske, 2018). KCs express toll-like receptor, which recognize molecules derived from microbes. Ligand–receptor combination changes their cytokine expression profiles. Differences in cytokine profiles greatly contribute to the development of specific skin diseases such as psoriasis and AD (Hänel et al., 2013; Noske, 2018; Zhang, 2019). Since these cytokines are thought to induce maturation of immune cells, which lead to further cytokine production, it is important to prevent the vicious cycle of cytokine networks in the treatment of disease.

**FIGURE 3:**
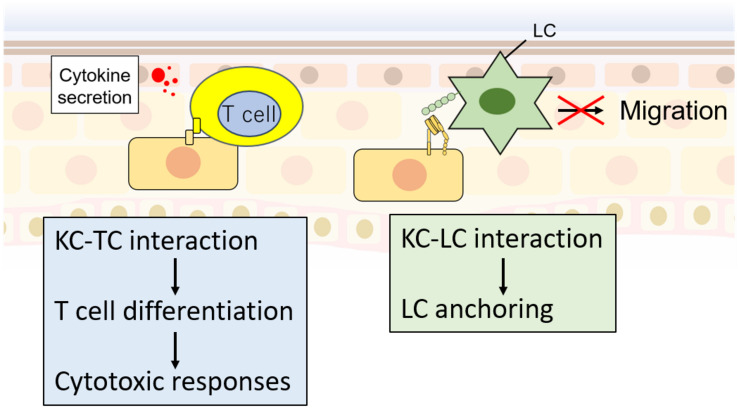
KC interaction with T cells (TCs) and LCs. KCs interact with TCs via CD2 and CD54, which promote TC differentiation. Differentiated TCs promote cytotoxic responses. In contrast, KC–LC interactions via E-cadherin and integrins decrease LC migration activity the to lymph node.

Some recent reports have shown that KCs interact with T cells (Banerjee et al., 2004; Peters et al., 2013; Albanesi et al., 2018; Orlik et al., 2020) and LCs (Tang et al., 1993; Mohammed et al., 2016; Sumpter et al., 2019) to regulate their immune responses (Figure 3). Since T cells and LCs play an important role in cytokine secretion and disease development, understanding their regulation mechanisms by KCs may provide novel insight into disease mechanisms. Orlik et al. (2020) reported that IFNγ-stimulated KCs could interact with naïve T cells through CD2 and CD54 to promote differentiation into proinflammatory Th1 and Th17 cells. Although this ability is low compared with monocytes or DCs (Orlik et al., 2020), this finding raises an important possibility that T cell differentiation by KCs is pathophysiologically important because KCs are much more abundant than APCs, including LCs, in the epidermis. In addition, interaction of CD4 or CD8 T cells with IL-1α/TNFα-stimulated KCs increased the secretion of CCL2, CCL20, and CXCL10 chemokines and increased Th17-like T cells (Peters et al., 2013). These reports suggest that the interaction between T cells and KCs may cause a Th17-dominant bias, leading to disruption of skin homeostasis (Figure 3). For the interaction between KC and LC, E-cadherin and integrins are required (Tang et al., 1993; Mohammed et al., 2016; Figure 3). Downregulation of αvβ6 integrins in KCs decreased epidermal LCs but increased lymph node LCs (Mohammed et al., 2016). This result suggests the function of KCs as the “anchor” of LCs, which might be important in regulating immune reactivity (Tang et al., 1993). Future investigation of how KCs regulate immune reactivity is required.

## Primary Cilia Function in Immunity

For a long time, it had been considered that immune cells have an immunological synapse (IS), instead of primary cilia (Cassioli and Baldari, 2019). IS is a ring structure containing adhesion molecules, T cell antigen receptor (TCR), Major Histocompatibility Complex (MHC), which mediate cell–cell interaction between APCs and lymphocytes (Cassioli and Baldari, 2019). IS promotes lymphocyte activation by sustaining signaling required for T cell activation (Finetti et al., 2011). The function of intraflagellar transport (IFT) protein IFT20 has been reported in immune synapse formation in T cells (Finetti et al., 2009; Finetti et al., 2011). Although IFTs are required for the assembly and maintenance of primary cilia, primary cilia in immune cells have been undetected until 2015. Prosser and Morrison (2015) reported that with regard to immortalized Jurkat T cell, and NALM-6 B cells had them at a low rate of 1% or less in the presence of serum. Recently, we found primary cilia-like structures in human primary LCs, which are APCs present in the epidermis (Toriyama et al., 2020). In contrast, we did not find such structures in epidermal or dermal CD4 + or CD8 + T cells. This finding raises the possibility that the tissue microenvironment or the extracellular environment controls primary cilia formation.

**FIGURE 4:**
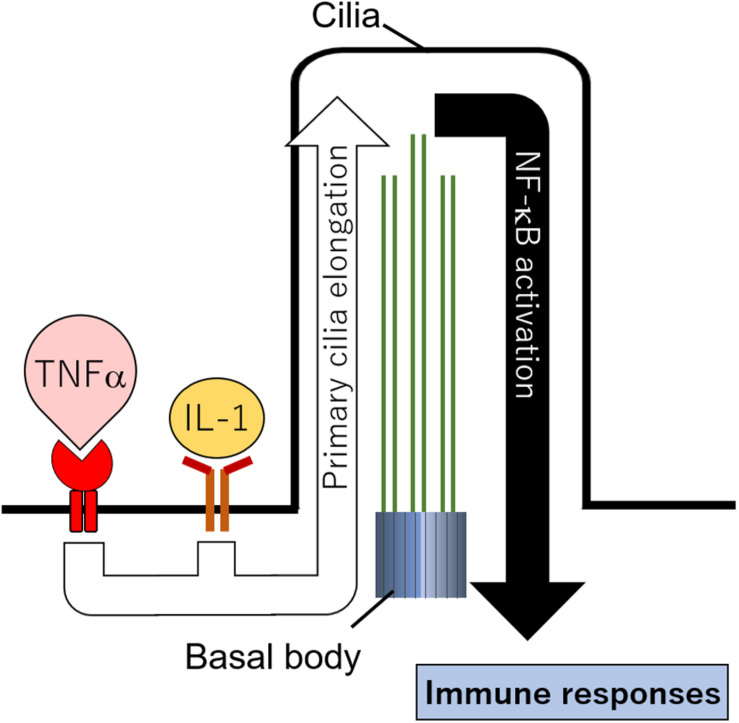
Hypothetical cilia regulation by immune signals. Cytokines including IL-1 and TNFα elongate primary cilia length. NFκB localized in primary cilia is activated and promotes immune responses.

So, what is the physiological role of primary cilia in immune cells? It is difficult to say because research to answer this question is scarce; however, we can say that primary cilia would have a role in promoting immune signaling. Interestingly, recent studies have reported that various immune signals promoted by cytokines regulate primary cilia formation in non-immune cells. Treatment with IL-1β or TNFα significantly increased the length of primary cilia in chondrocytes and fibroblasts (Wann and Knight, 2012; Wann et al., 2013; Mc Fie et al., 2020; Figure 4). Primary cilia were elongated 1 h after IL-1β treatment, suggesting that their length might change during acute inflammation (Wann et al., 2013). A mutation in tumor necrosis factor alpha receptor 3–interacting protein 1, also known as MIPT3, inhibited primary cilia formation (Berbari et al., 2011). MIPT3 inhibits IL-13–mediated phosphorylation of Stat6 (Ling and Goeddel, 2000; Niu et al., 2003). These findings strongly suggest that cytokine signals regulate primary cilia formation. In addition, promotion of iNOS and COX2 expression caused by IL-1β was decreased in chondrocytes with an *ift88* hypomorphic mutation, the Oak Ridge Polycystic Kidney (ORPK) mutation (Wann et al., 2014). In these cells, nuclear localization of p65, a transcription factor induced by NFκB signal activation, was decreased (Mc Fie et al., 2020). These findings suggest that IL-1β and TNFα elongate primary cilia, and NFκB signals activated by these cytokines are transduced in primary cilia (Figure 4). It should be noted, however, that there is no description of how the combination of Th1 and Th2 cytokines regulates primary cilia formation in inflammatory diseases. To answer this, further investigation using patient samples and cells will be required.

In ORPK mice with polycystic kidney disease (PKD), the number of infiltrating macrophages was increased, but the number of residential macrophages was decreased in renal lesions (Zimmerman et al., 2018). In human PKD patients, the number of T cells in lesions was also increased (Zimmerman et al., 2019). Further investigation using ORPK mice showed that bile duct epithelial cells increased the expression of C-C motif chemokine 2 (CCL2) (Zimmerman et al., 2018). Knockout of *CCR2*, a receptor for CCL2, in ORPK mice reduced numbers of infiltrating macrophages and PKD symptoms relative to those in ORPK single-dysfunction mice. Since primary cilia have not been found in macrophages, the regulation of macrophages by primary cilia is unknown; however, the importance of primary cilia signals transduced in epithelial cells that interact with immune cells and stroma cells to the regulation of macrophage infiltration is suggested (Zimmerman et al., 2018). Future studies need to elucidate the physiological role of primary cilia in immune cells during immune responses and the mechanism of primary cilia control by immune signals. Profiles of ciliary receptors/ion channels have not been identified in immune cells, so it is not clear what signals are transduced in the primary cilia in immune cells. As the signaling molecules in the primary cilia become clarified, the physiological role of primary cilia in immune cells will be elucidated.

**FIGURE 5:**
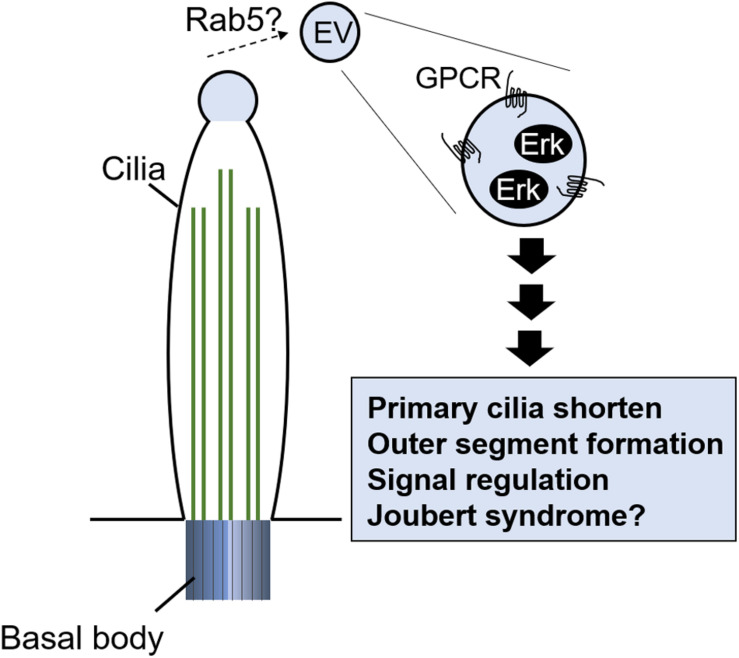
Potential of EVs released from primary cilia tip. EVs contain signal molecules including G protein–coupled receptor (GPCR) and Erk. Release of EVs regulates primary cilia length, outer segment formation in photoreceptor cells, and signal transduction.

Chargaff and West (1946) first described extracellular vesicles (EVs) in serum. EVs are cell-released vesicles, or ectosomes, surrounded by a lipid bilayer and contain various molecules: signaling proteins and enzymes, DNA, mRNA, and miRNA (Valadi et al., 2007). They are known to mediate intercellular signals and to control various cell activities, including immune response, early development of the embryo, and cancer progression (Latifkar et al., 2019; Margolis and Sadovsky, 2019). The physiological role of EVs released by immune cells has been studied in recent years; EVs released by APCs carry surface MHC class I and class II molecules and therefore directly activate CD4 + and CD8 + T cells. EVs derived from DCs, macrophages, and fibroblasts carry cytokines, including IL-1β and TNFα, which seem to have a role in mediating inflammatory and autoimmune diseases (see review Robbins and Morelli, 2014). The release of EVs is regulated by various mechanisms, but interestingly, primary cilia also play an important role in their production (Wood and Rosenbaum, 2015; Nager et al., 2017; Phua et al., 2017). What is the physiological role of ectosomes released from the primary ciliary tip? Ectosome release seems to be involved in the shortening of primary cilia associated with cell cycle progression (Phua et al., 2017), modulation of signal transduction by GPCR ectocytosis (Nager et al., 2017), and outer segment structure formation in photoreceptor cells (Salinas et al., 2017; Figure 5). Is there another function? To investigate the ectosome role, Zuo et al. (2019) identified the molecules in it by mass spectrometry. They found that Madin–Darby canine kidney (MDCK) cell–derived EVs contained MAPK regulating molecules, including Erk and phosphorylated (active) Erk (Zuo et al., 2019). The quantities of EVs are changed by primary cilia formation (Zuo et al., 2019), and a mutation in an exocyst protein, including RAB5, was found in Joubert syndrome, which features PKD due to ciliopathy (Dixon-Salazar et al., 2012). Ectosome formation and release into the extracellular space may contribute to tissue homeostasis, or to the development of disease, by regulating the MAPK pathway regulated by primary cilia and EV formation (Figure 5). Given the significant increase in primary cilia in AD (Toriyama et al., 2020), it is possible that the amount of ectosome derived from primary cilia may be altered in AD epidermis. Analysis using patient samples may reveal the physiological role of ectosomes derived from primary cilia.

## Relationship Between the Development of Skin Diseases and Primary Cilia

**FIGURE 6:**
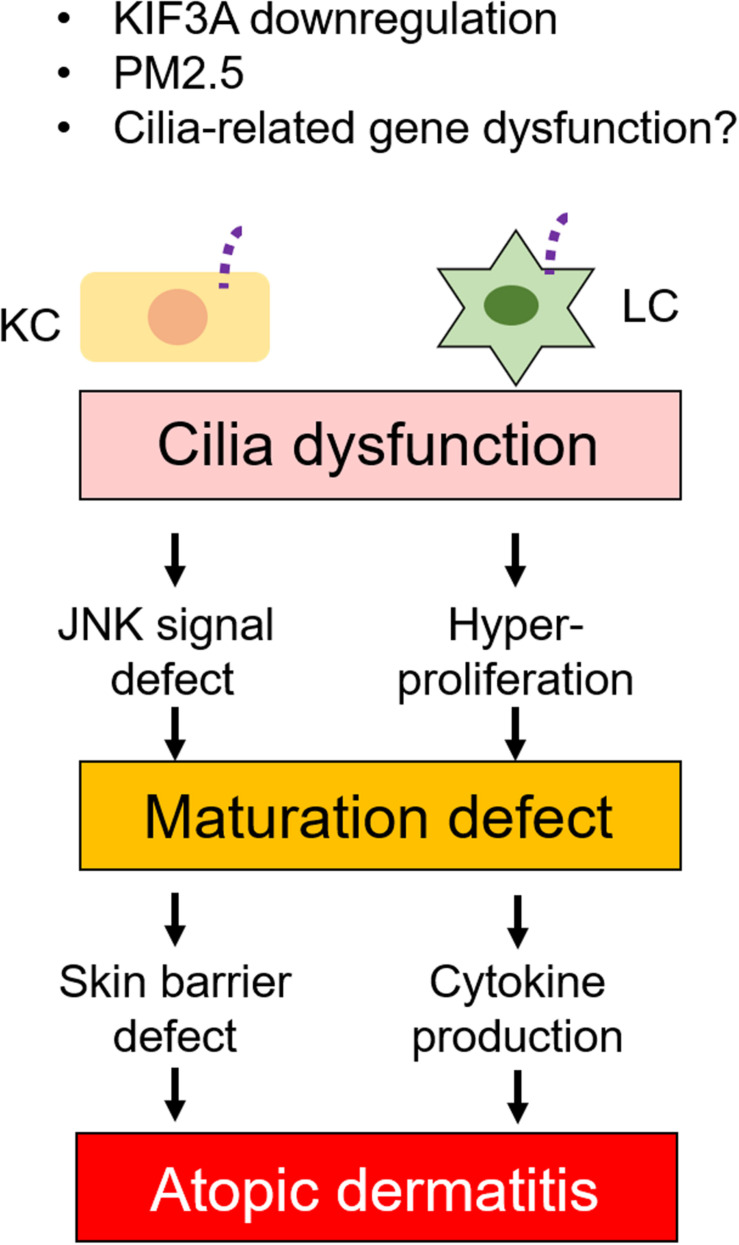
Relation between primary cilia defects and skin disease. Dysfunction of primary cilia caused by ciliary gene defects or immunostimulants changes KC and LC phenotypes. Cilia dysfunction causes JNK signal and hyperproliferation in KC and LC, respectively. These, in turn, cause maturation defect in both KC and LC. Maturation defect then causes skin barrier defect and cytokine production in KC and LC, respectively. Cilia regulation may have potential as a drug target.

Failure of skin immunity can lead to skin diseases. The skin immune response is excessive in AD, which affects up to 20% of children and up to 3% of adults (Nutten, 2015). Although the number of AD patients is increasing each year, only symptomatic treatment is currently used. As exacerbation and remission often repeat, details of the mechanism of AD onset and aggravation are required to develop therapeutic drugs (Eichenfield et al., 2014a,b; Katoh et al., 2020). Recently, one paper reported that particulate matters with aerodynamic diameter of 2.5 μm (PM2.5) inhibited ciliogenesis by increasing c-Jun expression in human KCs (Bae et al., 2019). PMs are known to cause AD, psoriasis, epithelium injury, eye injury, endothelial dysfunction, asthma, and chronic bronchitis with increasing inflammation (Gehring et al., 2010; Wang et al., 2012; Ahn, 2014; Hwang et al., 2016). Furthermore, the JNK pathway is known to be a critical element in inflammatory skin disease, as JNK is involved in multiple mechanisms that lead to gap junction and barrier protein defect (Hammouda et al., 2020). In this finding, Bae et al. did not investigate the direct relationship among inflammation caused by PM2.5, AD development, and primary ciliogenesis; however, they provided important insights that primary cilia signaling was necessary for the c-Jun pathway to regulate KC differentiation (Figure 6).

Interestingly, primary cilia-related genes, kinesin family member 3A (KIF3A), are related to AD development by modulating epidermal barrier functions (Stevens et al., 2020; Figure 6). Epidermal barrier formation is regulated by KC differentiation, and failure of the skin’s barrier function elevates the risk of developing AD (Sakai et al., 2015; Katoh et al., 2020). Since cornification is induced by mature KCs, filaggrin and loricrin are known as KC maturation markers. The expression of filaggrin and loricrin is decreased in 20–30% of AD patients, which causes disruption to barrier formation or cornification (Palmer et al., 2006; Kim et al., 2008; O’Regan et al., 2008). Single nucleotide polymorphisms (SNPs) in the *KIF3A* gene have been associated with AD, which decreased KIF3A in primary KCs from individuals (Stevens et al., 2020). Stevens et al. (2020) clearly demonstrated that KIF3A deficiency in mice caused skin barrier dysfunction. Even though they did not investigate primary cilia in donor skin, this finding suggests that primary cilia in KCs regulate differentiation and maturation balance, which is required for adequate skin barrier formation (Figure 6). Interestingly, the *KIF3A* gene was also identified as an asthma-related gene in childhood (Kovacic et al., 2011). Furthermore, ciliopathy patients frequently develop AD with other clinical features (Aldahmesh et al., 2014). In AD epidermis, when compared to healthy epidermis, the number of ciliated KCs and LCs were significantly increased and were immature (Toriyama et al., 2020). This finding raised the important possibility that primary cilia disruption was associated with allergic diseases, and it is required to investigate the pathophysiological function of other cilia-related genes in inflammatory diseases (Figure 6).

## Therapeutic Potential of Targeting Primary Cilia

Previous studies have suggested that abnormalities in primary cilia may be involved in the development of allergic diseases including AD (Aldahmesh et al., 2014; Bae et al., 2019; Stevens et al., 2020; Toriyama et al., 2020). Therefore, is it possible to treat allergic diseases by regulating primary cilia? Ciliobrevin A and chloral hydrate have been reported as agents that shorten primary cilia formation, while lithium chloride and folic acid have been reported as agents that extend them (Praetorius and Spring, 2003; Firestone et al., 2012; Thompson et al., 2016; Toriyama et al., 2017). Inhibitors of the folate metabolism pathway are widely used as anticancer agents and immunosuppressive agents to treat rheumatoid arthritis and severe asthma (Dyer et al., 1991; Singh et al., 2016; Koźmiński et al., 2020). Recent findings suggest that cytokines regulate primary ciliogenesis (Berbari et al., 2011; Wann and Knight, 2012; Wann et al., 2013; Mc Fie et al., 2020). Currently, the development of monoclonal antibody drugs targeting cytokines and cytokine receptors is progressing, and their numbers, both in use and in clinical trials, are increasing every year (Deleanu and Nedelea, 2019; Kamata and Tada, 2020). They may contribute to the suppression of inflammation by suppressing primary cilia formation.

However, even if primary cilia are directly involved in the control of inflammation, the control of primary cilia formation itself could be problematic on account of strong side effects because the primary cilia contain many signaling molecules, including receptors and channels, which would affect various cellular responses. Targeting specific molecules localized in primary cilia may contribute to the development of therapeutic agents.

Currently, the signal via the primary cilia that controls immune regulation is not well understood, but suppression of EP4, TRPV4, or PDGFRα, which are localized in primary cilia, may offer one therapeutic target (Kabashima et al., 2003; Schneider et al., 2005; Jin et al., 2014; Wang et al., 2019; Toriyama et al., 2020). As EP4 antagonist impaired contact hypersensitivity of skin with decreasing LC activation, it is one of the promising therapeutic candidates. Adjusting the number of LCs would be important in treatment because LCs are involved in cytokine/chemokine production and in exacerbation of disease (Yoshiki et al., 2014). However, proliferation and maturation mechanism of LCs are not fully understood, so it is necessary to understand the mechanisms of proliferation of LCs in the steady state, in the inflammatory state, and in disease (Merad et al., 2002; Seré et al., 2012; Collin and Milne, 2016). It is important to note that imatinib, a specific inhibitor of PDGFRα that is used for the treatment of leukemia, causes edema and keratosis pilaris as side effects (Leong and Aw, 2016). It is necessary to elucidate how this happens.

Taken together, identifying new signaling molecules in primary cilia will require the development of novel therapies and drugs to regulate immune responses. This will need a comprehensive search for primary cilia-localized receptors in various cell, tissue, and disease models.

## Concluding Remarks

Research to uncover the relationship between primary cilia regulation and immunoregulation has progressed in recent years and has brought new insights in the fields of immunology, physiology, cell biology, and pathology. What types of immune cells have primary cilia? What diseases are involved in primary cilia regulation? What are the characteristic features of ciliated cells? What signals are transduced in primary cilia? What is the physiological function of primary cilia *in vitro*? Answering these questions will contribute to new knowledge in biology and to drug discovery.

## Author Contributions

MT and KI contributed to the writing and editing of the manuscript. Both authors contributed to the article and approved the submitted version.

## Conflict of Interest

The authors declare that the research was conducted in the absence of any commercial or financial relationships that could be construed as a potential conflict of interest.

## References

[B1] Ahn K. (2014). The role of air pollutants in atopic dermatitis. *J. Allergy Clin. Immunol.* 134 993–999. 10.1016/j.jaci.2014.09.023 25439225

[B2] Albanesi C. Madonna S. Gisondi P. Girolomoni G. (2018). The interplay between keratinocytes and immune cells in the pathogenesis of psoriasis. *Front. Immunol.* 9:1549. 10.3389/fimmu.2018.01549 30034395 PMC6043636

[B3] Aldahmesh M. A. Li Y. Alhashem A. Anazi S. Alkuraya H. Hashem M. (2014). IFT27, encoding a small GTPase component of IFT particles, is mutated in a consanguineous family with Bardet-Biedl syndrome. *Hum. Mol. Genet.* 23 3307–3315. 10.1093/hmg/ddu044 24488770 PMC4047285

[B4] Bae J.-E. Choi H. Shin D. W. Na H.-W. Park N. Y. Kim J. B. (2019). Fine particulate matter (PM2.5) inhibits ciliogenesis by increasing SPRR3 expression via c-Jun activation in RPE cells and skin keratinocytes. *Sci. Rep.* 9:3994. 10.1038/s41598-019-40670-y 30850686 PMC6408442

[B5] Banerjee G. Damodaran A. Devi N. Dharmalingam K. Raman G. (2004). Role of keratinocytes in antigen presentation and polarization of human T lymphocytes. *Scand. J. Immunol.* 59 385–394. 10.1111/j.0300-9475.2004.01394.x 15049782

[B6] Bennett C. L. van Rijn E. Jung S. Inaba K. Steinman R. M. Kapsenberg M. L. (2005). Inducible ablation of mouse Langerhans cells diminishes but fails to abrogate contact hypersensitivity. *J. Cell Biol.* 169 569–576. 10.1083/jcb.200501071 15897263 PMC2171694

[B7] Berbari N. F. Kin N. W. Sharma N. Michaud E. J. Kesterson R. A. Yoder B. K. (2011). Mutations in Traf3ip1 reveal defects in ciliogenesis, embryonic development, and altered cell size regulation. *Dev. Biol.* 360 66–76. 10.1016/j.ydbio.2011.09.001 21945076 PMC4059607

[B8] Beura L. K. Hamilton S. E. Bi K. Schenkel J. M. Odumade O. A. Casey K. A. (2016). Normalizing the environment recapitulates adult human immune traits in laboratory mice. *Nature* 532 512–516. 10.1038/nature17655 27096360 PMC4871315

[B9] Candi E. Schmidt R. Melino G. (2005). The cornified envelope: a model of cell death in the skin. *Nat. Rev. Mol. Cell Biol.* 6 328–340. 10.1038/nrm1619 15803139

[B10] Cassioli C. Baldari C. T. (2019). A ciliary view of the immunological synapse. *Cells* 8:789. 10.3390/cells8080789 31362462 PMC6721628

[B11] Chargaff E. West R. (1946). The biological significance of the thromboplastic protein of blood. *J. Biol. Chem.* 166 189–197. 20273687

[B12] Choi H. Shin J. H. Kim E. S. Park S. J. Bae I.-H. Jo Y. K. (2016). Primary cilia negatively regulate melanogenesis in melanocytes and pigmentation in a human skin model. *PLoS One* 11:e0168025. 10.1371/journal.pone.0168025 27941997 PMC5152889

[B13] Chorro L. Sarde A. Li M. Woollard K. J. Chambon P. Malissen B. (2009). Langerhans cell (LC) proliferation mediates neonatal development, homeostasis, and inflammation-associated expansion of the epidermal LC network. *J. Exp. Med.* 206 3089–3100. 10.1084/jem.20091586 19995948 PMC2806478

[B14] Christensen S. T. Morthorst S. K. Mogensen J. B. Pedersen L. B. (2017). Primary cilia and coordination of Receptor Tyrosine Kinase (RTK) and transforming growth factor β (TGF-β) signaling. *Cold Spring Harb. Perspect. Biol.* 9:a028167. 10.1101/cshperspect.a028167 27638178 PMC5453389

[B15] Clayton E. Doupé D. P. Klein A. M. Winton D. J. Simons B. D. Jones P. H. (2007). A single type of progenitor cell maintains normal epidermis. *Nature* 446 185–189. 10.1038/nature05574 17330052

[B16] Coelho P. A. Bury L. Shahbazi M. N. Liakath-Ali K. Tate P. H. Wormald S. (2015). Over-expression of Plk4 induces centrosome amplification, loss of primary cilia and associated tissue hyperplasia in the mouse. *Open Biol.* 5:150209. 10.1098/rsob.150209 26701933 PMC4703062

[B17] Collin M. Milne P. (2016). Langerhans cell origin and regulation. *Curr. Opin. Hematol.* 23 28–35. 10.1097/MOH.0000000000000202 26554892 PMC4685746

[B18] Croyle M. J. Lehman J. M. O’Connor A. K. Wong S. Y. Malarkey E. B. Iribarne D. (2011). Role of epidermal primary cilia in the homeostasis of skin and hair follicles. *Development* 138 1675–1685. 10.1242/dev.060210 21429982 PMC3074444

[B19] Czernielewski J. M. Demarchez M. (1987). Further evidence for the self-reproducing capacity of Langerhans cells in human skin. *J. Invest. Dermatol.* 88 17–20. 10.1111/1523-1747.ep12464659 3540136

[B20] Dai D. Zhu H. Wlodarczyk B. Zhang L. Li L. Li A. G. (2011). Fuz controls the morphogenesis and differentiation of hair follicles through the formation of primary cilia. *J. Invest. Dermatol.* 131 302–310. 10.1038/jid.2010.306 20962855

[B21] De Benedetto A. Kubo A. Beck L. A. (2012). Skin barrier disruption: a requirement for allergen sensitization? *J. Invest. Dermatol.* 132 949–963. 10.1038/jid.2011.435 22217737 PMC3279586

[B22] Deckers J. Hammad H. Hoste E. (2018). Langerhans cells: sensing the environment in health and disease. *Front. Immunol.* 9:93. 10.3389/fimmu.2018.00093 29449841 PMC5799717

[B23] Deleanu D. Nedelea I. (2019). Biological therapies for atopic dermatitis: an update. *Exp. Ther. Med.* 17 1061–1067. 10.3892/etm.2018.6989 30679974 PMC6327672

[B24] Dixon-Salazar T. J. Silhavy J. L. Udpa N. Schroth J. Bielas S. Schaffer A. E. (2012). Exome sequencing can improve diagnosis and alter patient management. *Sci. Transl. Med.* 4:138ra78. 10.1126/scitranslmed.3003544 22700954 PMC4442637

[B25] Dyer P. D. Vaughan T. R. Weber R. W. (1991). Methotrexate in the treatment of steroid-dependent asthma. *J. Allergy Clin. Immunol.* 88 208–212. 10.1016/0091-6749(91)90330-q 1880321

[B26] Eckert R. L. Efimova T. Dashti S. R. Balasubramanian S. Deucher A. Crish J. F. (2002). Keratinocyte survival, differentiation, and death: many roads lead to mitogen-activated protein kinase. *J. Investig. Dermatol. Symp. Proc.* 7 36–40. 10.1046/j.1523-1747.2002.19634.x 12518790

[B27] Eichenfield L. F. Tom W. L. Berger T. G. Krol A. Paller A. S. Schwarzenberger K. (2014a). Guidelines of care for the management of atopic dermatitis: section 2. Management and treatment of atopic dermatitis with topical therapies. *J. Am. Acad. Dermatol.* 71 116–132. 10.1016/j.jaad.2014.03.023 24813302 PMC4326095

[B28] Eichenfield L. F. Tom W. L. Chamlin S. L. Feldman S. R. Hanifin J. M. Simpson E. L. (2014b). Guidelines of care for the management of atopic dermatitis: section 1. Diagnosis and assessment of atopic dermatitis. *J. Am. Acad. Dermatol.* 70 338–351. 10.1016/j.jaad.2013.10.010 24290431 PMC4410183

[B29] Elofsson R. Andersson A. Falck B. Sjöborg S. (1981). The human epidermal melanocyte. A ciliated cell. *Acta Dermato Venereol.* 61 49–52.

[B30] Elofsson R. Andersson A. Falck B. Sjöborg S. (1984). The ciliated human keratinocyte. *J. Ultrastruct. Res.* 87 212–220. 10.1016/s0022-5320(84)80061-1 6085808

[B31] Eyerich S. Eyerich K. Traidl-Hoffmann C. Biedermann T. (2018). Cutaneous barriers and skin immunity: differentiating a connected network. *Trends Immunol.* 39 315–327. 10.1016/j.it.2018.02.004 29551468

[B32] Ezratty E. J. Pasolli H. A. Fuchs E. (2016). A Presenilin-2-ARF4 trafficking axis modulates Notch signaling during epidermal differentiation. *J. Cell Biol.* 214 89–101. 10.1083/jcb.201508082 27354375 PMC4932368

[B33] Ezratty E. J. Stokes N. Chai S. Shah A. S. Williams S. E. Fuchs E. (2011). A role for the primary cilium in Notch signaling and epidermal differentiation during skin development. *Cell* 145 1129–1141. 10.1016/j.cell.2011.05.030 21703454 PMC3135909

[B34] Finetti F. Paccani S. R. Riparbelli M. G. Giacomello E. Perinetti G. Pazour G. J. (2009). Intraflagellar transport is required for polarized recycling of the TCR/CD3 complex to the immune synapse. *Nat. Cell Biol.* 11 1332–1339. 10.1038/ncb1977 19855387 PMC2837911

[B35] Finetti F. Paccani S. R. Rosenbaum J. Baldari C. T. (2011). Intraflagellar transport: a new player at the immune synapse. *Trends Immunol.* 32 139–145. 10.1016/j.it.2011.02.001 21388881 PMC4060984

[B36] Firestone A. J. Weinger J. S. Maldonado M. Barlan K. Langston L. D. O’Donnell M. (2012). Small-molecule inhibitors of the AAA+ ATPase motor cytoplasmic dynein. *Nature* 484 125–129. 10.1038/nature10936 22425997 PMC3321072

[B37] Gao J. DeRouen M. C. Chen C.-H. Nguyen M. Nguyen N. T. Ido H. (2008). Laminin-511 is an epithelial message promoting dermal papilla development and function during early hair morphogenesis. *Genes Dev.* 22 2111–2124. 10.1101/gad.1689908 18676816 PMC2492752

[B38] Gehring U. Wijga A. H. Brauer M. Fischer P. de Jongste J. C. Kerkhof M. (2010). Traffic-related air pollution and the development of asthma and allergies during the first 8 years of life. *Am. J. Respir. Crit. Care Med.* 181 596–603. 10.1164/rccm.200906-0858OC 19965811

[B39] Hammouda M. B. Ford A. E. Liu Y. Zhang J. Y. (2020). The JNK signaling pathway in inflammatory skin disorders and cancer. *Cells* 9:857. 10.3390/cells9040857 32252279 PMC7226813

[B40] Hänel K. H. Cornelissen C. Lüscher B. Baron J. M. (2013). Cytokines and the skin barrier. *Int. J. Mol. Sci.* 14 6720–6745. 10.3390/ijms14046720 23531535 PMC3645662

[B41] Herrmann T. van der Hoeven F. Grone H.-J. Stewart A. F. Langbein L. Kaiser I. (2003). Mice with targeted disruption of the fatty acid transport protein 4 (Fatp 4, Slc27a4) gene show features of lethal restrictive dermopathy. *J. Cell Biol.* 161 1105–1115. 10.1083/jcb.200207080 12821645 PMC2173002

[B42] Honda T. Nakajima S. Egawa G. Ogasawara K. Malissen B. Miyachi Y. (2010). Compensatory role of Langerhans cells and langerin-positive dermal dendritic cells in the sensitization phase of murine contact hypersensitivity. *J. Allergy Clin. Immunol.* 125 1154–1156.e2. 10.1016/j.jaci.2009.12.005 20226508

[B43] Hsu Y.-C. Li L. Fuchs E. (2014). Transit-amplifying cells orchestrate stem cell activity and tissue regeneration. *Cell* 157 935–949. 10.1016/j.cell.2014.02.057 24813615 PMC4041217

[B44] Hwang S. H. Choi Y.-H. Paik H. J. Wee W. R. Kim M. K. Kim D. H. (2016). Potential importance of ozone in the association between outdoor air pollution and dry eye disease in South Korea. *JAMA Ophthalmol.* 134 503–510. 10.1001/jamaophthalmol.2016.0139 26967354

[B45] Jin D. Ni T. T. Sun J. Wan H. Amack J. D. Yu G. (2014). Prostaglandin signalling regulates ciliogenesis by modulating intraflagellar transport. *Nat. Cell Biol.* 16:841. 10.1038/ncb3029 PMC4154319 25173977

[B46] Kabashima K. Honda T. Ginhoux F. Egawa G. (2019). The immunological anatomy of the skin. *Nat. Rev. Immunol.* 19 19–30. 10.1038/s41577-018-0084-5 30429578

[B47] Kabashima K. Sakata D. Nagamachi M. Miyachi Y. Inaba K. Narumiya S. (2003). Prostaglandin E2-EP4 signaling initiates skin immune responses by promoting migration and maturation of Langerhans cells. *Nat. Med.* 9 744–749. 10.1038/nm872 12740571

[B48] Kamata M. Tada Y. (2020). Efficacy and safety of biologics for psoriasis and psoriatic arthritis and their impact on comorbidities: a literature review. *Int. J. Mol. Sci.* 21:1690 10.3390/ijms21051690 PMC7084606 32121574

[B49] Kaplan D. H. Jenison M. C. Saeland S. Shlomchik W. D. Shlomchik M. J. (2005). Epidermal langerhans cell-deficient mice develop enhanced contact hypersensitivity. *Immunity* 23 611–620. 10.1016/j.immuni.2005.10.008 16356859

[B50] Katoh N. Ohya Y. Ikeda M. Ebihara T. Katayama I. Saeki H. (2019). Clinical practice guidelines for the management of atopic dermatitis 2018. *J. Dermatol.* 46 1053–1101. 10.1111/1346-8138.15090 31599013

[B51] Katoh N. Ohya Y. Ikeda M. Ebihara T. Katayama I. Saeki H. (2020). Japanese guidelines for atopic dermatitis 2020. *Allergol. Int.* 69 356–369. 10.1016/j.alit.2020.02.006 32265116

[B52] Kim B. E. Leung D. Y. Boguniewicz M. Howell M. D. (2008). Loricrin and involucrin expression is down-regulated by Th2 cytokines through STAT-6. *Clin. Immunol.* 126 332–337. 10.1016/j.clim.2007.11.006 18166499 PMC2275206

[B53] Kissenpfennig A. Malissen B. (2006). Langerhans cells–revisiting the paradigm using genetically engineered mice. *Trends Immunol.* 27 132–139. 10.1016/j.it.2006.01.003 16458606

[B54] Klar J. Schweiger M. Zimmerman R. Zechner R. Li H. Törmä H. (2009). Mutations in the fatty acid transport protein 4 gene cause the ichthyosis prematurity syndrome. *Am. J. Hum. Genet.* 85 248–253. 10.1016/j.ajhg.2009.06.021 19631310 PMC2725242

[B55] Klareskog L. Tjernlund U. Forsum U. Peterson P. A. (1977). Epidermal Langerhans cells express Ia antigens. *Nature* 268 248–250. 10.1038/268248a0 329142

[B56] Kovacic M. B. Myers J. M. B. Wang N. Martin L. J. Lindsey M. Ericksen M. B. (2011). Identification of KIF3A as a novel candidate gene for childhood asthma using RNA expression and population allelic frequencies differences. *PLoS One* 6:e23714. 10.1371/journal.pone.0023714 21912604 PMC3166061

[B57] Kowalevsky A. (1867). “Entwickelungsgeschichte des *Amphioxus lanceolatus*,” in *Proceedings of the Memoires de l’Academie Imperiale des Sciences*, St.-Petersbourg.

[B58] Koźmiński P. Halik P. K. Chesori R. Gniazdowska E. (2020). Overview of Dual-acting drug methotrexate in different neurological diseases, autoimmune pathologies and cancers. *Int. J. Mol. Sci.* 21:3483. 10.3390/ijms21103483 32423175 PMC7279024

[B59] Kubo A. Nagao K. Yokouchi M. Sasaki H. Amagai M. (2009). External antigen uptake by Langerhans cells with reorganization of epidermal tight junction barriers. *J. Exp. Med.* 206 2937–2946. 10.1084/jem.20091527 19995951 PMC2806471

[B60] Langerhans P. (1868). Ueber die nerven der menschlichen haut. *Arch. Pathol. Anat. Physiol. Klin. Med.* 44 325–337. 10.1007/BF01959006

[B61] Latifkar A. Hur Y. H. Sanchez J. C. Cerione R. A. Antonyak M. A. (2019). New insights into extracellular vesicle biogenesis and function. *J. Cell Sci.* 132 222406. 10.1242/jcs.222406 31263077 PMC6633391

[B62] Lechler T. Fuchs E. (2005). Asymmetric cell divisions promote stratification and differentiation of mammalian skin. *Nature* 437 275–280. 10.1038/nature03922 16094321 PMC1399371

[B63] Lehman J. M. Laag E. Michaud E. J. Yoder B. K. (2009). An essential role for dermal primary cilia in hair follicle morphogenesis. *J. Invest. Dermatol.* 129 438–448. 10.1038/jid.2008.279 18987668 PMC2677658

[B64] Leong W. M. S. Aw C. W. D. (2016). Nilotinib-induced Keratosis Pilaris. *Case Rep. Dermatol.* 8 91–96. 10.1159/000445676 27194977 PMC4868941

[B65] Ling L. Goeddel D. V. (2000). MIP-T3, a novel protein linking tumor necrosis factor receptor-associated factor 3 to the microtubule network. *J. Biol. Chem.* 275 23852–23860. 10.1074/jbc.M001095200 10791955

[B66] Mackenzie I. C. (1970). Relationship between mitosis and the ordered structure of the stratum corneum in mouse epidermis. *Nature* 226 653–655. 10.1038/226653a0 5444930

[B67] Margolis L. Sadovsky Y. (2019). The biology of extracellular vesicles: the known unknowns. *PLoS Biol.* 17:e3000363. 10.1371/journal.pbio.3000363 31318874 PMC6667152

[B68] Mascré G. Dekoninck S. Drogat B. Youssef K. K. Broheé S. Sotiropoulou P. A. (2012). Distinct contribution of stem and progenitor cells to epidermal maintenance. *Nature* 489 257–262. 10.1038/nature11393 22940863

[B69] Mc Fie M. Koneva L. Collins I. Coveney C. R. Clube A. M. Chanalaris A. (2020). Ciliary proteins specify the cell inflammatory response by tuning NFκB signalling, independently of primary cilia. *J. Cell Sci.* 133:jcs239871. 10.1242/jcs.239871 32503942 PMC7358134

[B70] Merad M. Manz M. G. Karsunky H. Wagers A. Peters W. Charo I. (2002). Langerhans cells renew in the skin throughout life under steady-state conditions. *Nat. Immunol.* 3 1135–1141. 10.1038/ni852 12415265 PMC4727838

[B71] Mohammed J. Beura L. K. Bobr A. Astry B. Chicoine B. Kashem S. W. (2016). Stromal cells control the epithelial residence of DCs and memory T cells by regulated activation of TGF-β. *Nat. Immunol.* 17 414–421. 10.1038/ni.3396 26901152 PMC5135085

[B72] Nager A. R. Goldstein J. S. Herranz-Pérez V. Portran D. Ye F. Garcia-Verdugo J. M. (2017). An actin network dispatches ciliary GPCRs into extracellular vesicles to modulate signaling. *Cell* 168 252–263.e14. 10.1016/j.cell.2016.11.036 28017328 PMC5235987

[B73] Nakajima S. Igyártó B. Z. Honda T. Egawa G. Otsuka A. Hara-Chikuma M. (2012). Langerhans cells are critical in epicutaneous sensitization with protein antigen via thymic stromal lymphopoietin receptor signaling. *J. Allergy Clin. Immunol.* 129 1048–1055.e6. 10.1016/j.jaci.2012.01.063 22385635 PMC4600611

[B74] Nestle F. O. Di Meglio P. Qin J.-Z. Nickoloff B. J. (2009). Skin immune sentinels in health and disease. *Nat. Rev. Immunol.* 9 679–691. 10.1038/nri2622 19763149 PMC2947825

[B75] Niu Y. Murata T. Watanabe K. Kawakami K. Yoshimura A. Inoue J. (2003). MIP-T3 associates with IL-13Ralpha1 and suppresses STAT6 activation in response to IL-13 stimulation. *FEBS Lett.* 550 139–143. 10.1016/s0014-5793(03)00860-3 12935900

[B76] Noske K. (2018). Secreted immunoregulatory proteins in the skin. *J. Dermatol. Sci.* 89 3–10. 10.1016/j.jdermsci.2017.10.008 29111181

[B77] Nutten S. (2015). Atopic dermatitis: global epidemiology and risk factors. *Ann. Nutr. Metab.* 66(Suppl. 1), 8–16. 10.1159/000370220 25925336

[B78] O’Regan G. M. Sandilands A. McLean W. H. I. Irvine A. D. (2008). Filaggrin in atopic dermatitis. *J. Allergy Clin. Immunol.* 122 689–693. 10.1016/j.jaci.2008.08.002 18774165

[B79] Orlik C. Deibel D. Küblbeck J. Balta E. Ganskih S. Habicht J. (2020). Keratinocytes costimulate naive human T cells via CD2: a potential target to prevent the development of proinflammatory Th1 cells in the skin. *Cell. Mol. Immunol.* 17 380–394. 10.1038/s41423-019-0261-x 31324882 PMC7109061

[B80] Palmer C. N. Irvine A. D. Terron-Kwiatkowski A. Zhao Y. Liao H. Lee S. P. (2006). Common loss-of-function variants of the epidermal barrier protein filaggrin are a major predisposing factor for atopic dermatitis. *Nat. Genet.* 38 441–446. 10.1038/ng1767 16550169

[B81] Pasparakis M. Haase I. Nestle F. O. (2014). Mechanisms regulating skin immunity and inflammation. *Nat. Rev. Immunol.* 14 289–301. 10.1038/nri3646 24722477

[B82] Peters J. H. Tjabringa G. S. Fasse E. de Oliveira V. L. Schalkwijk J. Koenen H. J. P. M. (2013). Co-culture of healthy human keratinocytes and T-cells promotes keratinocyte chemokine production and RORγt-positive IL-17 producing T-cell populations. *J. Dermatol. Sci.* 69 44–53. 10.1016/j.jdermsci.2012.10.004 23127421

[B83] Phua S. C. Chiba S. Suzuki M. Su E. Roberson E. C. Pusapati G. V. (2017). Dynamic remodeling of membrane composition drives cell cycle through primary cilia excision. *Cell* 168 264–279.e15. 10.1016/j.cell.2016.12.032 28086093 PMC5660509

[B84] Phua S. C. Lin Y.-C. Inoue T. (2015). An intelligent nano-antenna: primary cilium harnesses TRP channels to decode polymodal stimuli. *Cell Calcium* 58 415–422. 10.1016/j.ceca.2015.03.005 25828566 PMC4564334

[B85] Praetorius H. A. Spring K. R. (2003). Removal of the MDCK cell primary cilium abolishes flow sensing. *J. Membr. Biol.* 191 69–76. 10.1007/s00232-002-1042-4 12532278

[B86] Prosser S. L. Morrison C. G. (2015). Centrin2 regulates CP110 removal in primary cilium formation. *J. Cell Biol.* 208 693–701. 10.1083/jcb.201411070 25753040 PMC4362459

[B87] Rerknimitr P. Otsuka A. Nakashima C. Kabashima K. (2017). The etiopathogenesis of atopic dermatitis: barrier disruption, immunological derangement, and pruritus. *Inflamm. Regen.* 37:14. 10.1186/s41232-017-0044-7 29259713 PMC5725646

[B88] Robbins P. D. Morelli A. E. (2014). Regulation of immune responses by extracellular vesicles. *Nat. Rev. Immunol.* 14 195–208. 10.1038/nri3622 24566916 PMC4350779

[B89] Roshan A. Murai K. Fowler J. Simons B. D. Nikolaidou-Neokosmidou V. Jones P. H. (2016). Human keratinocytes have two interconvertible modes of proliferation. *Nat. Cell Biol.* 18 145–156. 10.1038/ncb3282 26641719 PMC4872834

[B90] Sakai T. Hatano Y. Zhang W. Fujiwara S. Nishiyori R. (2015). Knockdown of either filaggrin or loricrin increases the productions of interleukin (IL)-1α, IL-8, IL-18 and granulocyte macrophage colony-stimulating factor in stratified human keratinocytes. *J. Dermatol. Sci.* 80 158–160. 10.1016/j.jdermsci.2015.09.002 26381575

[B91] Salinas R. Y. Pearring J. N. Ding J.-D. Spencer W. J. Hao Y. Arshavsky V. Y. (2017). Photoreceptor discs form through peripherin-dependent suppression of ciliary ectosome release. *J. Cell Biol.* 216 1489–1499. 10.1083/jcb.201608081 28381413 PMC5412563

[B92] Schneider L. Clement C. A. Teilmann S. C. Pazour G. J. Hoffmann E. K. Satir P. (2005). PDGFRalphaalpha signaling is regulated through the primary cilium in fibroblasts. *Curr. Biol.* 15 1861–1866. 10.1016/j.cub.2005.09.012 16243034

[B93] Schou K. B. Pedersen L. B. Christensen S. T. (2015). Ins and outs of GPCR signaling in primary cilia. *EMBO Rep.* 16 1099–1113. 10.15252/embr.201540530 26297609 PMC4576980

[B94] Schuler G. Steinman R. M. (1985). Murine epidermal Langerhans cells mature into potent immunostimulatory dendritic cells in vitro. *J. Exp. Med.* 161 526–546. 10.1084/jem.161.3.526 3871837 PMC2187584

[B95] Seré K. Baek J.-H. Ober-Blöbaum J. Müller-Newen G. Tacke F. Yokota Y. (2012). Two distinct types of Langerhans cells populate the skin during steady state and inflammation. *Immunity* 37 905–916. 10.1016/j.immuni.2012.07.019 23159228

[B96] Shimizu H. (2018). *Atarashii Hifukagaku*, 3rd Edn, Tokyo: Nakayamashoten.

[B97] Simpson C. L. Patel D. M. Green K. J. (2011). Deconstructing the skin: cytoarchitectural determinants of epidermal morphogenesis. *Nat. Rev. Mol. Cell Biol.* 12 565–580. 10.1038/nrm3175 21860392 PMC3280198

[B98] Singh J. A. Saag K. G. Bridges S. L. Jr. Akl E. A. Bannuru R. R. Sullivan M. C. (2016). 2015 American college of rheumatology guideline for the treatment of rheumatoid arthritis. *Arthrit. Care Res.* 68 1–25. 10.1002/acr.22783 26545825

[B99] Stevens M. L. Zhang Z. Johansson E. Ray S. Jagpal A. Ruff B. P. (2020). Disease-associated KIF3A variants alter gene methylation and expression impacting skin barrier and atopic dermatitis risk. *Nat. Commun.* 11:4092. 10.1038/s41467-020-17895-x 32796837 PMC7427989

[B100] Stingl G. Wolff-Schreiner E. C. Pichler W. J. Gschnait F. Knapp W. Wolff K. (1977). Epidermal Langerhans cells bear Fc and C3 receptors. *Nature* 268 245–246. 10.1038/268245a0 887158

[B101] Strugnell G. E. Wang A. M. Wheatley D. N. (1996). Primary cilium expression in cells from normal and aberrant human skin. *J. Submicrosc. Cytol. Pathol.* 28 215–225. 8964046

[B102] Sumpter T. L. Balmert S. C. Kaplan D. H. (2019). Cutaneous immune responses mediated by dendritic cells and mast cells. *JCI Insight* 4:e123947. 10.1172/jci.insight.123947 30626752 PMC6485911

[B103] Tang A. Amagai M. Granger L. G. Stanley J. R. Udey M. C. (1993). Adhesion of epidermal Langerhans cells to keratinocytes mediated by E-cadherin. *Nature* 361 82–85. 10.1038/361082a0 8421498

[B104] Thompson C. L. Wiles A. Poole C. A. Knight M. M. (2016). Lithium chloride modulates chondrocyte primary cilia and inhibits hedgehog signaling. *FASEB J. Off. Publ. Fed. Am. Soc. Exp. Biol.* 30 716–726. 10.1096/fj.15-274944 26499268

[B105] Toriyama M. Rizaldy D. Nakamura M. Fujita F. Okada F. Morita A. (2020). Immunological role of primary cilia of dendritic cells in human skin disease. *bioRxiv* [Preprint], 10.1101/2020.02.04.933333

[B106] Toriyama M. Toriyama M. Wallingford J. B. Finnell R. H. (2017). Folate-dependent methylation of septins governs ciliogenesis during neural tube closure. *FASEB J. Off. Publ. Fed. Am. Soc. Exp. Biol.* 31 3622–3635. 10.1096/fj.201700092R 28432198 PMC5503710

[B107] Valadi H. Ekström K. Bossios A. Sjöstrand M. Lee J. J. Lötvall J. O. (2007). Exosome-mediated transfer of mRNAs and microRNAs is a novel mechanism of genetic exchange between cells. *Nat. Cell Biol.* 9 654–659. 10.1038/ncb1596 17486113

[B108] Wang T. Wang L. Moreno-Vinasco L. Lang G. D. Siegler J. H. Mathew B. (2012). Particulate matter air pollution disrupts endothelial cell barrier via calpain-mediated tight junction protein degradation. *Part Fibre Toxicol.* 9:35. 10.1186/1743-8977-9-35 22931549 PMC3489700

[B109] Wang Z. Zhou L. An D. Xu W. Wu C. Sha S. (2019). TRPV4-induced inflammatory response is involved in neuronal death in pilocarpine model of temporal lobe epilepsy in mice. *Cell Death Dis.* 10:386. 10.1038/s41419-019-1612-3 31097691 PMC6522539

[B110] Wann A. K. Chapple J. P. Knight M. M. (2014). The primary cilium influences interleukin-1beta-induced NFkappaB signalling by regulating IKK activity. *Cell Signal* 26 1735–1742. 10.1016/j.cellsig.2014.04.004 24726893 PMC4064300

[B111] Wann A. K. Knight M. M. (2012). Primary cilia elongation in response to interleukin-1 mediates the inflammatory response. *Cell Mol. Life Sci.* 69 2967–2977. 10.1007/s00018-012-0980-y 22481441 PMC3417094

[B112] Wann A. K. Thompson C. L. Chapple J. P. Knight M. M. (2013). Interleukin-1β sequesters hypoxia inducible factor 2α to the primary cilium. *Cilia* 2:17. 10.1186/2046-2530-2-17 24330727 PMC3886195

[B113] Wong R. Geyer S. Weninger W. Guimberteau J.-C. Wong J. K. (2016). The dynamic anatomy and patterning of skin. *Exp. Dermatol.* 25 92–98. 10.1111/exd.12832 26284579

[B114] Wong S. Y. Seol A. D. So P.-L. Ermilov A. N. Bichakjian C. K. Epstein E. H. J. (2009). Primary cilia can both mediate and suppress Hedgehog pathway-dependent tumorigenesis. *Nat. Med.* 15 1055–1061. 10.1038/nm.2011 19701205 PMC2895420

[B115] Wood C. R. Rosenbaum J. L. (2015). Ciliary ectosomes: transmissions from the cell’s antenna. *Trends Cell Biol.* 25 276–285. 10.1016/j.tcb.2014.12.008 25618328 PMC4409478

[B116] Yamamoto H. Hattori M. Chamulitrat W. Ohno Y. Kihara A. (2020). Skin permeability barrier formation by the ichthyosis-causative gene FATP4 through formation of the barrier lipid ω-O-acylceramide. *Proc. Natl. Acad. Sci. U.S.A.* 117 2914–2922. 10.1073/pnas.1917525117 31974308 PMC7022171

[B117] Yoshiki R. Kabashima K. Honda T. Nakamizo S. Sawada Y. Sugita K. (2014). IL-23 from Langerhans cells is required for the development of imiquimod-induced psoriasis-like dermatitis by induction of IL-17A-producing γδ T cells. *J. Invest. Dermatol.* 134 1912–1921. 10.1038/jid.2014.98 24569709

[B118] Zhang L.-J. (2019). Type1 interferons potential initiating factors linking skin wounds with psoriasis pathogenesis. *Front. Immunol.* 10:1440. 10.3389/fimmu.2019.01440 31293591 PMC6603083

[B119] Zimmerman K. A. Gonzalez N. M. Chumley P. Chacana T. Harrington L. E. Yoder B. K. (2019). Urinary T cells correlate with rate of renal function loss in autosomal dominant polycystic kidney disease. *Physiol. Rep.* 7:e13951. 10.14814/phy2.13951 30632307 PMC6328912

[B120] Zimmerman K. A. Song C. J. Gonzalez-Mize N. Li Z. Yoder B. K. (2018). Primary cilia disruption differentially affects the infiltrating and resident macrophage compartment in the liver. *Am. J. Physiol. Gastrointest. Liver Physiol.* 314 G677–G689. 10.1152/ajpgi.00381.2017 29543508 PMC6048441

[B121] Zingg D. Debbache J. Peña-Hernández R. Antunes A. T. Schaefer S. M. Cheng P. F. (2018). EZH2-mediated primary cilium deconstruction drives metastatic melanoma formation. *Cancer Cell* 34 69–84.e14. 10.1016/j.ccell.2018.06.001 30008323

[B122] Zuo X. Kwon S.-H. Janech M. G. Dang Y. Lauzon S. D. Fogelgren B. (2019). Primary cilia and the exocyst are linked to urinary extracellular vesicle production and content. *J. Biol. Chem.* 294 19099–19110. 10.1074/jbc.RA119.009297 31694916 PMC6916495

